# Survey data linking coastal visit behaviours to socio-demographic and health profiles

**DOI:** 10.1038/s41597-024-03161-y

**Published:** 2024-03-27

**Authors:** Alexander Hooyberg, Henk Roose, Britt Lonneville, Stefaan De Henauw, Nathalie Michels, Gert Everaert

**Affiliations:** 1 Flanders Marine Institute (VLIZ), Ostend, Belgium; 2 Department of Sociology, Ghent University (UGent), Ghent, Belgium; 3 Department of Public Health and Primary Care, Ghent University (UGent), Ghent, Belgium; 4 Department of Developmental, Personality and Social Psychology, Ghent University (UGent), Ghent, Belgium

**Keywords:** Culture, Therapeutics, Sociology, Psychology, Epidemiology

## Abstract

Coastal destinations are highly popular for leisure, yet the effects of spending time at the coast on mental and physical health have remained underexplored. To accelerate the research about the effects of the coast on health, we compiled a dataset from a survey on a sample (N = 1939) of the adult Flemish population about their visits to the Belgian coast. The survey queried the number of days spent at the coast in the previous year or before and the following characteristics of their visits: how often they performed specific activities, which of the 14 municipal seaside resorts they visited, who they were with, what they mentally and physically experienced, and what reasons they had for not visiting the coast more often. The respondents’ geo-demographic (including residential proximity to the coast), socio-economic, and health profile was also collected. We anticipate that investigations on the data will increase our understanding about the social structuring of coastal visits and give context to the effects of the coast on human health.

## Background & Summary

More than half of all tourism involves a coastal or marine destination, and blue tourism annually accounts for 4.6 trillion US dollar or 5.2% of the global gross domestic product^1^. Coastal destinations have been attractive for centuries because of their beneficial effects on mental and physical health^2–5^. Previous research found that spending time near the ocean reduces stress^6–8^, promotes physical activity^9,10^, and provides a setting to meet with family and friends^11,12^. It is now clear that residing in closer proximity to the coast and visiting the coast more often effectuates these benefits, similarly as what has been evidenced for inland blue and green spaces^13–16^. However, it is still unknown to which extent the characteristics of the coastal visit and visitor modify the health outcomes.

**Fig. 1:**
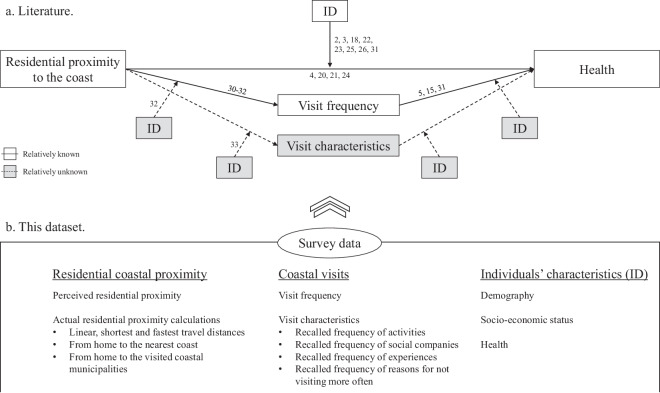
Overview of what this dataset contributes to the current literature. Panel a shows a conceptual diagram of the mediating roles of visit frequency and visit characteristics and the moderating role of individual traits (ID) in explaining the benefits of residential coastal proximity for health. A distinction is made between what is known from the literature and what this data provides to complement existing knowledge. Panel b shows what this dataset contains.

Studies from across the globe provided the initial evidence for the beneficial effects of the coast by revealing that residing in closer proximity to the coast is associated with a better self-reported general and mental health (e.g., in Belgium^4^, Canada^17^, China^18^, Ireland^19,20^, Japan^21^, Spain^22^, and the United Kingdom^2,3,23–25^). Several reviews and conceptual frameworks hypothesize that this pattern occurs because people who live nearer the coast tend to visit it more often^16,26–28^ (Fig. 1a). Indeed, cross-country analyses have confirmed that living nearer the coast is associated with a higher coastal visit frequency^29,30^ and that a higher coastal visit frequency is associated with a better self-reported general health^5,15,30^. It also seemed that these pathways are moderated by the demographic and socio-economic characteristics and health of the individual^16,26–28^. For example, Boyd *et al*.^31^ clearly illustrated for England that “infrequent users [of coastal environments] were more likely to be female, older, in poor health, of lower socioeconomic status, of ethnic minority status, live in relatively deprived areas with less neighbourhood greenspace and be further from the coast”. However, examples from outside England are now required to strengthen our understanding about how individual characteristics moderate the coastal visit frequency and experienced health effects.

Next to the visit frequency, the visit characteristics may be an equally important mechanism by which residential proximity benefits general health. Coastal visitors perform many different leisure activities at the coast^32^, and each activity may result in distinct emotional, cognitive, and physical experiences that contribute to overall health^33^. Depending on the individual’s socio-demography and health, different activities may be performed and different health effects may be experienced^32^. Unfortunately, no study seems to have yet investigated who performs what kinds of recreational activities at the coast, and whether these different activities result in different experiences and health effects (Fig. 1a).

## Knowledge Gap

There is a lack of high-quality scientific data that links individual characteristics such as residential proximity, demography, socio-economic status, and health to the coastal visit frequency and visit characteristics and the resulting emotional, cognitive, and physical experiences. Coastal nations’ tourism agencies usually do survey the activities, reasons, and experiences with the coastal amenities alongside the demographic features of their coastal visitors. However, in many cases, crucial variables such as residential coastal proximity, socio-economic status, and what people experienced emotionally and cognitively are neglected, because collecting the data often merely must serve the optimization of blue tourism. England seems to be an exception, because there the Monitor of Engagement with the Natural Environment (MENE) survey queries where people go, what they do, and what they experienced at the coast, alongside age, sex, deprivation indexes, and other characteristics of the individuals^34^. This data has proven to be very effective for science, because it has led to significant advancements in the current knowledge about coastal recreation and health relationships^13,31,32,35,36^.

### Purpose of this dataset

The aim of this research was to develop a dataset that allows to perform confirmatory and exploratory investigations on the relationships between residential proximity to the coast and the resulting mental and physical experiences via the mediating effects of the visit frequency and characteristics, and the moderating role of the individuals’ socio-demography and health (Fig. 1b). Therefore, a survey was distributed among Flemish-speaking Belgian inhabitants and their visits to the Belgian coast (i.e. not international coastal tourism). We deliberately focused on a local scale to be able to reveal a diversity of relationships and patterns within the locally-specific cultural landscape. The data contains the visit frequency, visited municipal seaside resorts, performed activities, gained experiences, and applicable reasons for not visiting the coast more often alongside the geographic (i.e. postal codes), demographic, socio-economic, and health profiles of the respondents. We supplemented this data with the objective linear distances, shortest, and fastest driving distances from the centres of the home municipalities to the nearest and actually visited municipal seaside resorts, similarly as in previous research^2,4^. The dataset holds complete responses from 1939 respondents, of whom 1304 had visited the Belgian coast in the preceding year (67.25%), 627 had not visited the coast in the preceding year but did before (32.34%), and eight respondents had never visited the Belgian coast (0.41%). For the users’ convenience, each variable in the raw and processed data is given a detailed description in a codebook that is shared along with the data (folder ‘3. Processing’).

The dataset may be of interest for researchers aiming to disentangle relationships between the ocean and human health and for stakeholders of blue tourism^37–43^. More specifically, the data allows to unravel the social structuring of recreational activities to the coast, which can be analysed via multivariate modelling or ordination techniques. Alternatively, the data can also contribute to a number of ongoing investigations in the literature, and we propose thematic research questions that may be addressed with the data: about ‘coastal epidemiology and accessibility’, ‘health and psycho-physical experiences’, ‘social relations’, and ‘issues of time, season, and weather’ (Table 1). Four fields of application for the data are identified: to increase our understanding of the coastal recreation phenomenon; to help to address the needs, challenges, and opportunities in the blue tourism sector; to evaluate whether and how the coast can be used for new cost-effective health-care practices (e.g. coastal visits on prescription); and to help spatial planners to design the coast up to the needs of the residents and visitors. This publication provides univariate descriptions of the data, which can assist in shedding light on the variation present in the data and the quality of the data. By making this dataset publicly available and in accordance with FAIR principles, it also calls to researchers and tourism agencies to standardize coastal tourism questionnaires and make existing and newly acquired data openly available.

**Table 1:** A non-exhaustive list of thematic research questions that can be addressed with the proposed dataset. The themes may overlap with each other.

Potential uses of the proposed dataset
1) Unravelling the social structuring of coastal visits
i. Which demographic, socio-economic and health profiles are associated with which visit frequency and recreational activities, social companies, and experiences at the coast and with which reasons for not visiting the coast more often?
2) Contributing to ongoing investigations in the literature
• Coastal epidemiology and accessibility
i. Is living nearer the coast associated with a better self-reported general and mental health?
ii. Is living nearer the coast associated with more physical activity and social support?
iii. Is living nearer the coast associated with a higher coastal visit frequency?
iv. How well does the perceived residential proximity match with the actual residential proximity?
v. What are reasons for not visiting the coast more often?
vi. What municipal seaside resorts are visited for performing which coastal activities?
vii. Do coastal residents perform different kinds of activities and visit different seaside resorts than tourists from inland?
viii. How do these relations differ for people with a poorer mental and physical health or poorer socio-economic status?
• Health – Psycho-physical experiences
i. What experiences are gained from visiting the coast and do these experiences support attention restoration theory and psycho-evolutionary theory?
ii. How do the experiences at the coast link with the visit frequency and the performed activities?
iii. Do citizens who have a poorer health or chronic illnesses visit the coast more/less often and perform different activities compared to citizens who are healthy, and (how) do their experiences differ?
iv. Can visiting the coast minimize health disparities?
• Social relations
i. How does the household composition and level of social support relate to the visit frequency, the activities performed, and the social company during the coastal visits?
ii. How does the social company associate with particular experiences during the visit?
iii. How does summer crowding and off-season calmness affect visitors’ experiences and reasons for not visiting the coast more often?
• Season – Weather – Time
i. How are a person’s occupational status and time availability linked to the coastal visit frequency, in which season the coast is visited, and the reasons for not visiting the coast more often?
ii. How do the season in which the coast is visited and the different weather phenomena (e.g. wind, clouds, precipitation) associate with the experiences, and for who is the season/weather a reason for not visiting the coast more often?
iii. Does the visit frequency and residential proximity during childhood relate to the (nostalgic) experiences at the coast now?

## Methods

### Survey

The dataset contains the responses to an online survey about the performed recreational visits to the Belgian coast and the demographic, socio-economic, and health background of the respondents. The survey was distributed among a panel of 30.000 to 35.000 Flemish-speaking members from the five provinces in Flanders that had subscribed to participate in societally relevant research (Bpact, Leuven, Belgium). It was distributed from January 2^nd^ to January 17^th^ 2023 to meet the intended number of 1640 complete responses. Sampling happened via quota sampling based on data about the age (<34 y, 35–49 y, 50–64 y, 50–64 y and >65 y), sex, province, and educational attainment (categories: low, middle, high) that was previously gathered by the panel provider. Sampling happened during multiple waves while considering propensity scores per quota. Oversampling of quotas was allowed, and no exclusion criteria were set. In total, 2574 panellists responded to the survey, of whom 1939 provided a complete and reliable response (see section 4. Technical Validation). The respondents received points from the panel provider (quantity unknown for the researchers) for the time spent on the survey, and these points add up to an appropriate monetary compensation. The survey was anonymous and consent for voluntary participation was acquired via panel subscription. The research was conducted according to the ethical rules presented in the General Ethical Protocol of the Faculty of Psychology and Educational Sciences of Ghent University. The survey was administered in Dutch via the online Bpact user interface and Qualtrics software^44^. The survey itself can be found within the data^45^ in the folder ‘/1. Survey’ and the acquired responses in folder ‘/2. Raw data/a. Survey responses/’.

Asking about respondents’ socio-demographic background and health could lead to social desirability bias. To be able to assess potential measurement error, we asked at the end of the survey how comfortable the respondents were with answering each section of the survey (i.e. about coastal visits, demography, employment situation and income, and health) using a five-item multiple choice with answers ‘very discomfortable’, ‘discomfortable’, ‘neutral’, ‘comfortable’, and ‘very comfortable’^46^.

### Coastal visits

**Fig. 2:**
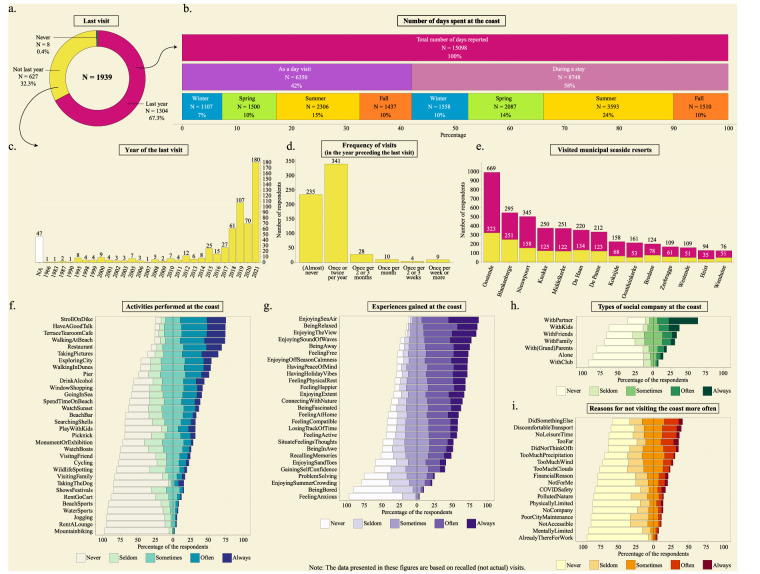
Descriptive graphs of the queried coastal visit frequency and coastal visit characteristics. Panels a to d show the acquired information about the visit frequency, panels e to i about the visit characteristics.

The questions about the coastal visits in the survey were designed to optimally capture the diversity of visit frequencies and characteristics. Furthermore, the questions meant to capture the respondents’ general perceptions and trends about many of their past coastal visits across seasons and years, rather than detailed information about only a couple of their visits (e.g., of visits in the last four weeks, as in previous research^15,29^). These general perceptions and trends were deemed to be more indicative for summarizing a respondent’s coastal visit behaviour and for distinguishing visit profiles across socio-demographic groups. Coastal visits were operationalized as days at which the person was at the Belgian coast in a recreational context and saw the sea. Depending on whether the respondents’ visited the Belgian coast in the previous year or before that, the reference period differed and additional questions about the frequency and locations of the performed visits were asked (Table 2). All respondents were asked to report how frequent 32 activities were (or would be) performed, how frequent the person was (or would be) accompanied by 7 types of social company, how frequent 27 experiences were (or would be) felt, and how frequent 18 reasons for not visiting the coast more often applied (or would apply; Table 2). Response categories for these questions were ‘never’, ‘seldom’, ‘sometimes’, ‘often’, and ‘always’. The items and response categories of the activities, types of social company, experiences, and reasons for not visiting the coast more often were chosen based on the local culture, the potential outcomes and mechanisms described in the nature and health literature^16,28,47,48^, previous studies about the experiences along the Belgian coast^49,50^, and the following previous surveys: the ‘Monitor of Engagement with the Natural Environment’ survey being administered nationally in England (MENE)^32,34^, the ‘Cultural participation in Flanders’ surveys being administered yearly in Flanders (from 1996 as the ‘SCV-survey’^51–53^, and since 2019 as part of Flanders’ ‘SV-survey’), and the surveys administered to day visitors and stayers in coastal accommodations by the local tourism agency aimed at informing policy^54,55^. Thus, we did not blindly copy item sets that were available in the literature, but rather designed our own based on our perspectives on the current knowledge. The respondents who visited the coast in the previous year also had to report for each of the four seasons within that year how many days the person was at the coast during a coastal day visit or during a multi-day visit with overnight stay in an accommodation at the coast. They also had to report which of the 14 municipal seaside resorts were visited. The respondents who had not visited the coast in the previous year but did before had to additionally report the frequency of coastal visits in that period, and which of the 14 municipal seaside resorts were then visited. Figure 2 displays the variation in the responses with regard to the coastal visits.

**Table 2:** Overview of the questions about the visits to the Belgian coast that were asked to the respondents who visited the Belgian coast in the previous year, to those who did not visit the Belgian coast in the previous year but visited the Belgian coast before that, and to those who never visited the Belgian coast.

Categorization questions	Have you visited the Belgian coast in the previous year?		
	Answer: Yes	Answer: No	
		Have you ever visited the Belgian coast?	
		Answer: Yes	Answer: No
Reference	Referring to the previous year (Jan. 1^st^ 2022 to Dec. 31^st^ 2022)	Referring to the last time(s) that the individual visited the Belgian coast	Referring to a hypothetical scenario in which the individual would visit the coast
Question topics	14 Municipal seaside resorts	14 Municipal seaside resorts	/
	Days and stays per season in the previous year	Year of the last visit	/
		Frequency of coastal visits in the year preceding the last visit	
	32 Activities	32 Activities	32 Activities
	7 Types of social company	7 Types of social company	7 Types of social company
	27 Experiences	27 Experiences	27 Experiences
	18 Reasons for not visiting the coast more often	18 Reasons for not visiting the coast more often	18 Reasons for not visiting the coast more often

**Fig. 3:**
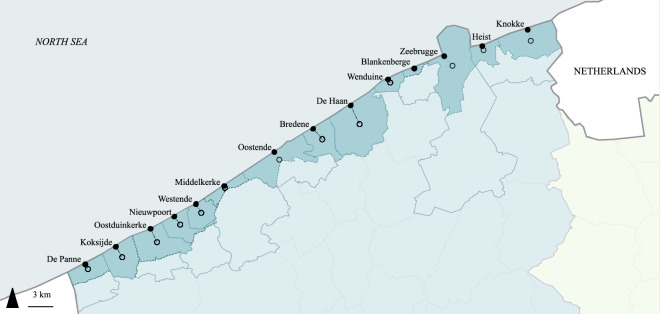
Map of the Belgian coast with municipal seaside resorts queried in the survey. Municipality borders are delineated by a solid line, the borders of sub-municipalities to which the seaside resorts belong by a dashed line. Open black circles represent the centroids of the polygons of the municipal seaside resorts, solid black circles the modelled destinations.

Note that the Belgian coast consists of 10 administrative municipalities, but the names that Belgian citizens commonly give to the 14 municipal seaside resorts often differ from the administrative boundaries (Table 3, Fig. 3).

**Table 3:** The names of the municipal seaside resorts as queried in the survey and the administrative sub-municipalities to which they respectively refer to.

Name of the municipal seaside resort as queried in the survey	Administrative sub-municipality(/-ies) to which the municipal seaside resort belongs to	Administrative municipality to which the sub-municipality(/-ies) belong(s) to
Blankenberge	Blankenberge	Blankenberge
Bredene	Bredene	Bredene
De Haan	Klemskerke + Vlissegem	De Haan
De Panne	De Panne	De Panne
Heist	Heist	Knokke-Heist
Knokke	Knokke	Knokke-Heist
Koksijde	Koksijde	Koksijde
Middelkerke	Middelkerke	Middelkerke
Nieuwpoort	Nieuwpoort	Nieuwpoort
Oostduinkerke	Oostduinkerke	Koksijde
Oostende	Oostende	Oostende
Wenduine	Wenduine	Wenduine
Westende	Westende + Lombardsijde	Middelkerke
Zeebrugge	Lissewege	Brugge

### Demographic background

**Fig. 4:**
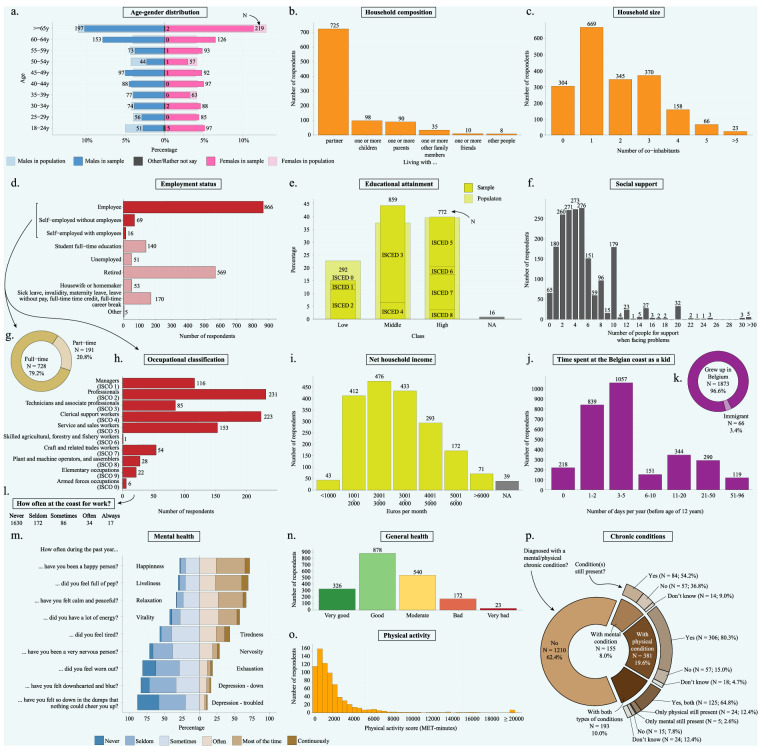
Descriptive graphs of the demography, socio-economic status, and health of the respondents. Panels a, b, c, f, j, and k show the demographic and social context of the respondents, panels d, e, g, h, i, and l show the socio-economic context, and panels m, n, o, and p their health and physical activity.

The section in the survey about the demographic background aimed to capture the life stage and living situation in physical and social space. The queried traits of the respondents were the following: year of birth, gender, the postal code of the primary residence, four optional postal codes of secondary residence locations, whether the respondent had a secondary residence at the coast that is sometimes visited for leisure, the perceived residential distance to the nearest coastline, the number and types of co-inhabitants, the number of people on which the respondent can count on when faced with serious problems (this self-designed proxy for social support is a simplified version of a previously-published single-item questionnaire for social support^56^ and hints to the same constructs as a multi-item social support questionnaire^57^), whether and where the person grew up in Belgium, and how often the coast was visited during childhood. The user can link these data to the frequency and characteristics of the coastal visits to evaluate the influence of geography, the social context, and mechanisms of nostalgia – an emotion that has proven to be crucial when investigating coastal visits and experiences^50,58^. Figure 4 panels a, b, c, f, j, and k visualize the variation in these demographic parameters. Some respondents’ postal codes (1000 and 1090) were in the Brussels-Capital Region (N = 2) and were not meant to be sampled via the panel, but we kept these respondents in the data for completeness.

### Socio-economic status

The survey included different proxies for the respondents’ socio-economic status. Firstly, the educational attainment was queried using the descriptions of the nine main categories in the International Standard Classification of Education (ISCED 0 to 8). There was also an ‘other’ category where respondents could specify their educational degree in case of uncertainty, but this resulted in some unclear responses that were identified as ‘NA’. Secondly, the employment situation distinguished the active (i.e. employee, self-employed with and without employees, student full-time education) from non-active (i.e. unemployed, retired, housewife or homemaker, out due to sickness or other circumstances) population using a multiple-choice question with one possible answer. There was also an ‘other’ category. The employees and self-employed respondents were also asked about their employment time (i.e. working full-time or part-time) and their occupation using the first-order and second-order classifications of the International Standard Classification of Occupations (ISCO). The survey also queried how often the respondent’s occupation involved being at a coastal or marine environment to evaluate the potential of constrained restoration^59–62^. Lastly, all respondents were asked about their net household income using increments of thousands (i.e., <1000 euros/month, 1001–2000 euros/month, …, >6001 euros/month). Figure 3 panels d, e, g, h, I, and i visualise the variation in the socio-economic responses.

### Health

Information about the health of the respondents was gathered for three reasons. First, it could help evaluate whether coastal visit behaviours are moderated by a person’s mental and physical health, for example in cases of limited mobility or depressive symptoms with a tendency towards social isolation. Second, it could help identify whether particular coastal visit behaviours, such as visiting the coast more often or performing particular coastal activities, are associated with a better or worse health as an outcome. Thirdly, it could provide further support for the relationship between living nearer the coast and self-reported general health or other proxy for health^4^. The questions related to the health of the respondents included the self-reported general health (first item from the short-form health survey, SF1), aspects of mental health (mental health part of the short-form health survey, SF36MH), including items referring to arousal/vitality (liveliness, vitality, exhaustion, tiredness) and to the valence/emotionality (nervosity, depression – feeling troubled, depression – feeling down, happiness), and having been diagnosed with a mental or physical chronic condition and whether this condition is still present. Also questioned were the time spent doing light, moderate, and intense physical activity in the past month (international physical activity questionnaire short form; IPAQ-SF)^63^. Figure 3 panels m-p visualize the variation in the health of the respondents.

### Nature connectedness

The survey was closed with a one-item question about the respondents’ nature connectedness, stating “Do you rather agree or disagree with the following sentence? Like a tree can be part of a forest, I feel embedded within the broader natural world”. Answers categories were ‘totally agree’, ‘agree’, ‘neutral’, ‘disagree’, ‘totally disagree’. This question is one item from the connectedness to nature scale that has shown to be particularly indicative of nature connectedness based on item response theory^64^.

### Processing steps

The processing steps with regard to the survey data can be found in Tables 4 and 5.

**Table 4:** Listed questions for the coastal visit characteristics in the survey and the type of answers and processing steps. Besides the mentioned processing steps, every variable was also translated from Dutch to English.

Category	Question topic	Answer type	Processing steps
Coastal visits	Visited coast in the previous year	Yes/No	Used to distinguish three types of visitors (visited last year, visited not last year but before, never visited)
	Ever visited the coast	Yes/No	
	Visited municipal seaside resorts	Multiple choice	For each seaside resort dichotomized into Yes/No
	Day visits to the coast in winter, spring, summer, and fall in the previous year	Numerical input	Number of days at the coast summed per season, per day visits, and per longer stays; Number of days topped off at theoretical maximum quantities
	Days at the coast during longer stays in winter, spring, summer, and fall in the previous year	Numerical input	
	Year of the last visit	Numerical input	Years >2021 set to NA*
	Frequency of coastal visits in the year preceding the last visit	Single choice	/
	Frequency of performed activities	Single choice	/
	Frequency of types of social company	Single choice	/
	Frequency of experiences	Single choice	/
	Frequency of reasons for not visiting the coast more often	Single choice	/

* Some respondents stated to not have visited the coast in 2022 but indicated the year of their last visit was 2022 (N = 34) or 2023 (N = 3). These values were set to ‘not available’ (NA).

**Table 5:** Listed questions for the individual characteristics in the survey and the type of answers and processing steps. Besides the mentioned processing steps, every variable was also translated from Dutch to English.

Category	Question topic	Answer type	Processing steps
Demography	Birth year	Numerical input	Used to derive the age
	Gender	Single choice	/
	Postal code(s) of residence (one primary and four optional secondary postal codes)	Numerical input	Cleaned*
	Perceived residential proximity to the coast	Single choice	/
	Grew up in Belgium	Yes/No	/
	Postal code of residence at age 5–15 years (one primary and four optional secondary postal codes)	Numerical input	Cleaned*
	Perceived average number of days per year at the coast at age <12 years	Numerical input	Topped off at 365
	Number of co-inhabitants	Numerical input	/
	Types of co-inhabitants	Multiple choice	Corrected choice ‘other’; Dichotomized
	Social support	Numerical input	/
	Having a holiday residence at the Belgian coast	Yes/No	/
Socio-economic status	Educational attainment (ISCED)	Categorical	Assigned ISCED codes; Corrected choice ‘other’
	Employment situation	Single choice	Corrected choice ‘other’; Dichotomized
	Employment full/half time	Single choice	/
	Occupation (ISCO)	Single choice	Assigned ISCO codes; Corrected choice ‘other’
	Frequency of being at the coast for work	Single choice	/
	Net household income	Single choice	/
Health	Number of minutes of light, moderate, and high intensity physical activity per week (IPAQ-SF)	Numerical input	Each topped off at theoretical maximum quantities, then multiplied by METs and summed
	Self-reported general health (SF1)	Single choice	/
	Diagnosed with a chronic mental condition	Yes (specify)/No	Specifications categorized
	Chronic mental condition still present	Yes/No	/
	Diagnosed with a chronic physical condition	Yes (specify)/No	Specifications categorized
	Chronic physical condition still present	Yes/No	/
	Mental health (Short Form 36 mental health)	Single choice	Average scores calculated for all items, and items of vitality and emotional health
Closing questions	Comfort of responding to questions about coastal visits, demography, employment situation and income, and health	Single choice	/
	Connectedness to nature	Single choice	/

* Some postal codes did not match with an existing administrative (sub-)municipality and were set to ‘not available’ (NA). Space characters were neglected, and postal codes with more or less than four digits or that contained non-numeric characters were set to ‘not available’ (NA).

### Additional calculations

#### Weights

The quota sampling inevitably caused an imperfect representativeness of the sample for the population. To clarify these sampling errors and correct for them to a possible degree, post-stratification weights were calculated for different strata based on the combinations of age (18–29 y, 30–39 y, 40–49 y, 50–59 y, 60–64 y, >= 65 y, total), sex (male, female, total), educational attainment (low, middle, high, total), and province (West Flanders, East Flanders, Antwerp, Limburg, Flemish Brabant, total). For each stratum, the weight was calculated as the frequency of individuals that belong to the stratum in the population divided by the frequency of individuals that belong to the stratum in the sample. Population statistics were retrieved from Statbel^65^. The number of strata were reduced to retain the specificity of the strata while limiting the number of excessively high or low weights due to exceptionally under- or oversampled strata, respectively. More specifically, consecutive age categories that were either both under- or both over-represented by the sample were pooled together (Fig. 4 panel a), so that the deviations from representativeness did not cancel out during the pooling. Since the population data contained sex data, and not gender data as in the survey, we had to consider the gender categories ‘Male’ and ‘Female’ to be matching the sexes ‘Male’ and ‘Female’ from the population. This gender-sex linkage and the different meanings of these concepts may have resulted in misrepresentative weights. Educational attainment was pooled into ‘Low’, ‘Middle’, and ‘High’, because the quota sampling also adopted these categories and this considerably decreased the number of excessively high weights. The Province categories were not adjusted to retain geographical specificity for each weight. If information for these four parameters were incomplete for an individual (e.g. NA for Educational attainment), those parameters were disregarded and the weight was calculated based on the parameters for which information was available. There were 504 strata with different combinations of age, gender-sex, educational attainment, and province categories. The weights were trimmed at 0.2 and 5. The population statistics retrieved from Statbel^65^ can be found within the data^45^ in the folder ‘/2. Raw data/b. Population socio-demographics/’. The weights table with pooled ISCED and age categories as described above is stored within the data^45^ on location ‘/3. Processing/R workspace/Weights.ISCEDLMH.Age.csv’. All weights were appended to the final processed survey data.

#### Residential proximity to the coast

**Fig. 5:**
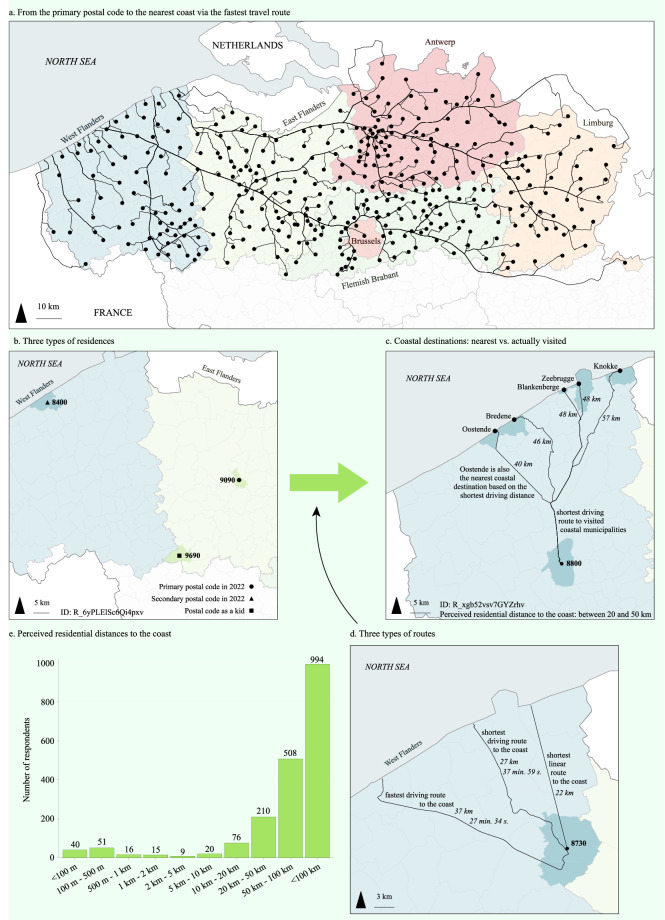
Visualization of the proxies for residential proximity to the coast. Panel a displays the sampled municipalities and the fastest driving routes to the coast. Panel b shows an example of three types of residences of a respondent. Panel c illustrates that the distance can be calculated to the nearest coast and to the actually visited seaside resorts from the primary postal code in 2022 from a respondent. Panel d displays an example of the different types of routes and associated distances and travel times to the nearest coast. Panel e shows the summary of the respondents’ perceived residential distance to the nearest coast.

Residential proximity to the coast was operationalized in different ways depending on the type of residence (primary and secondary; in 2022 and as a child), the destination (nearest coast vs. visited seaside resorts), and the route between the two (linear, shortest and fastest driving route; Fig. 5). Firstly, the distances were calculated from all residential primary and secondary postal codes in 2022 and as a child to the nearest coast. Secondly, the distances were calculated from the residential primary and secondary postal codes in 2022 to the actually visited seaside resorts in that year. As such, the user of this dataset can choose the proxy that is best suited for quantifying the residential proximity to the coast. For each distance, the residential municipality centroids were used as starting point. The distances corresponding with the linear and shortest and fastest driving routes were derived in kilometres, and for the shortest and fastest driving routes also in the number of seconds travel time. The destination points of the visited seaside resorts were the points along the shorelines that were closest to the centroids of the municipal seaside resorts (Fig. 3). The routes and accompanying distances were calculated with geographical information system (GIS)-methods: QuantumGIS 3.2.2 was used to generate a map with the OpenStreetMap road network (OpenStreetMap contributors, 2018) and Eurostat coastline data (Nomenclature of Territorial Units for Statistics (NUTS), 2013) and the ArcGIS Pro 2.2.0 Network Analyst extension was used to generate the routes and calculate the distances. Next to these objective measures of residential proximity, the perceived residential proximity to the nearest coast (in kilometres) was also queried in the survey. The raw distances calculated via GIS can be found within the data^45^ in the folder ‘/2. Raw data/c. Residential distances to the coast/’. The distances to the nearest coast were appended to the survey data with R^66^, which is stored at ‘/4. Processed data/’ in the files ‘Hooyberg_Survey_Processed.csv’ and ‘Hooyberg_Survey_Processed.txt’. The distances to the destinations were stored in a separate dataset due to its long format in the folder ‘/4. Processed data/’ in the files ‘Hooyberg_Distances_Processed.csv’ and ‘Hooyberg_Distances_Processed.txt’. The dataset also holds a merged dataset with all survey data and distances under the folder ‘/4. Processed data’/ in files ‘Hooyberg_Survey_Distances_Processed.csv’ and ‘Hooyberg_Survey_Distances_Processed.txt’.

## Data Records

To provide the reader with maximal transparency about the questioning of the items in the survey and the processing steps implemented for the final datasets, the zipped data contains four folders with the survey itself, the raw data, the processing process, the processed data, and the figures. All data can be found within the data^45^. The zipped file is 161 megabytes in size and the unzipped file 372 megabytes.

### Folder ‘1. Survey’

The first folder provides the survey in different formats and languages. The survey was administered in Dutch using the Qualtrics survey administration software, and English translations were added in Qualtrics later for publication.

- The file ‘Hooyberg_SurveyCoastalVisits_NL.docx’ is the exported human-readable format of the survey with all the rules and flows of the survey in the original language Dutch.
- The file ‘Hooyberg_SurveyCoastalVisits_ENG.docx’ is the same but with the English translation (translation was done after administration).
- The file ‘Hooyberg_SurveyCoastalVisits_NL_print.pdf’ clearly visualizes the layout and how the survey was shown to the respondents.
- The file ‘Hooyberg_SurveyCoastalVisits_Qualtrics.qsf’ is the Qualtrics project that can be loaded into the software.
- The file ‘Hooyberg_SurveyCoastalVisits_translations_NL-EN.csv’ contains the Dutch to English translations of the survey, which was downloaded from Qualtrics and which can be uploaded again if translations would have been lost in the Qualtrics project.

### Folder ‘2. Raw data’

The raw data folder contains the raw survey responses (folder ‘a. Survey responses’), the population statistics from which the weights were calculated (folder ‘b. Population socio-demographics’), and the proxies for residential proximity to the coast derived by GIS (folder ‘c. Residential distances to the coast’).

#### Folder ‘a. Survey responses’

The folder ‘a. Survey responses’ contains the complete and incomplete responses that were downloaded from Qualtrics at the end of the survey administration on January 18^th^ 2023. Each folder has the responses in different formats: comma separated values format (.csv), a format to be loaded in to IBM SPSS Statistics (.sav), tab-separated values format (.tsv), MS Excel standard format (.xlsx), and the MS Excel-compatible extensible mark-up language format (.xml).

#### Folder ‘b. Population socio-demographics’

The folder ‘b. Population socio-demographics’ contains the original population statistics per stratum as originally received by Statbel (file ‘Hooyberg_Pop_2023-08-01-Statbel.xlsx’) and the re-formatted data in wide (file ‘Hooyberg_Pop_2023-08-10_wide.xlsx’) and long (file ‘Hooyberg_Pop_2023-08-10_wide.xlsx’) format to be loaded into R.

#### Folder ‘c. Residential distances to the coast’

The different proxies for residential proximity to the coast are stored in two files. The first file (‘Hooyberg_DistancesToNearestCoast.csv’) contains a list of all the residential postal codes reported by the respondents and the corresponding distances to the nearest coast. The second file (‘Hooyberg_DistancesToDestinations.csv’) contains for each respondent the distances from the different types of residences to the visited seaside resorts. Both files were later merged with the survey data (see also section Methods – Additional calculations - ‘Residential proximity to the coast’).

### Folder ‘3. Processing’

The processing folder contains the codebook and the folder ‘R workspace’. The codebook (‘Hooyberg_SurveyCoastalVisits_Codebook.csv’) describes all of the original and newly added variables in the data with their coded names and formats. The folder ‘R workspace’ contains the latest version of the R script (named ‘Hooyberg_SurveyCoastalVisits_2023-08-31.R’), the weights calculated from the population and sample statistics per stratum (‘Weights.ISCEDLMH.Age.csv’, see section Methods – Additional calculations - Weights), and the postal codes for the municipal seaside resorts (‘Seaside_resorts_ZIP.csv’). The R workspace folder also contains the folder ‘Adjustments’. This folder contains help files for correcting respondents’ erroneous answers to the ‘other’ answer categories for household company (‘Household_Company_other_adjustments.csv’), education level (‘Education_other_adjustments.csv’), and occupational employment (‘Employment_other_adjustments.csv’). The folder ‘Adjustments’ also contains the categorisations and translations of the chronic mental and physical illnesses reported by the respondents (‘Chronics_mental_and_physical_illness_translation.csv’). Lastly, the ‘Adjustments’ folder contains two files that were used to identify the invalid responses by speeding and/or straightlining. A first file contains all the complete responses (N = 1949) and was used to visually scroll through the data to search for patterns of speeding and/or straight-lining (‘Survey_speeders_straightliners_highlighted.xlsx’). A second file contains the ID’s of these invalid responses (N = 10) that were loaded into R for exclusion from the data (‘Survey_speeders_straightliners_IDs.csv’).

### Folder ‘4. Processed data’

The folder ‘4. Processed data’ harbours the final survey data in which the distances to the nearest coast are embedded (files ‘Hooyberg_Survey_Processed.csv’ and ‘Hooyberg_Survey_Processed.txt’). It also contains the final distances data to the visited seaside resorts (files ‘Hooyberg_Distances_Processed.csv’ and ‘Hooyberg_Distances_Processed.csv’). Both of these datasets were also merged together (file ‘Hooyberg_Survey_Distances_Processed.csv’ and ‘Hooyberg_Survey_Distances_Processed.txt’).

### Folder ‘5. Figures’

The folder ‘5. Figures’ contains the original figures included in this descriptor and their individual panels. The panels were imported in Adobe Illustrator (file ‘Hooyberg_Survey_Descriptive_Graphs_2022-08-31.ai’) and their format was clarified and made consistent for resulting in the final files for Fig. 2, Fig. 4, and Fig. 5. The folder ‘Raw figures from GIS’ holds Fig. 3 and all the original panels of Fig. 5 that were generated via GIS. The folder ‘Raw figures from GIS’ also holds a figure overviewing all the primary and secondary residential postal codes in 2022 and those as a child. The folder ‘Raw figures from R’ contains the original panels of Fig. 2 and Fig. 4 that were generated and exported in R based on the survey data.

## Technical Validation

### Representativeness for the Flanders’ population

The use of an access panel greatly increased the representativeness of the Flanders population compared to convenience or similar other survey sampling methods. It also ensured that all invited panellists were blinded for the survey topic to reduce selection-bias. However, the sample was not perfectly representative for the population based on age, gender/sex, educational attainment, and province of residence. In general, the majority of the strata were oversampled and the majority of the sampled individuals (77.6%) belonged to an oversampled stratum with a weight less than 1. This was to be expected from the quota sampling procedure with oversampling allowance performed by the chosen panel provider. As a result, the data contains an overrepresentation of individuals that are 60-to-64-year-old, 40-to-49-year-old, belong to the middle socio-economic class, and reside in another province than Flemish Brabant (Fig. 4 panels a and e). Underrepresented are the individuals that are 50-to-59-year-old, of lower socio-economic class, and who reside in Flemish Brabant (Fig. 4 panels a and e). Also interesting was that 1 stratum had a weight of lower or equal than 0.2 that was assigned to 42 respondents (2.17%). Twenty-five strata had a weight higher or equal than 5 that was assigned to 5 respondents (0.26%). The data provided by the panel provider was insufficient to calculate design or non-response weights. It is difficult to compare the data about the coastal visits with other sources (e.g. by tourism agencies) because of the different sampling designs, different reference periods, and units of measurement.

### Response quality

All original questions (in Dutch) with their best possible English translations can be found within the data (folder ‘1. Survey’), and any issues with regard to the quality of the responses can be attributed to the manner of asking the questions^46,67^.

Only complete and valid responses were retained (N = 1939). Responses were regarded as complete when the last question of the survey was answered (N = 1949). Respondents that subsequently not proceeded to the end page or to the BPact panel user interface (N = 86) were retained, but note that these responses were not considered during the quota sampling and may have resulted in disproportional oversampling of the quotas (but see section Methods – Additional calculations – Weights and section Technical Validation – Representativeness for the Flanders’ population). Responses with relatively quick answering patterns (‘speeders’) and with repeated similar – often contradictory – answers (e.g. ‘always’ on all of the performed activities; ‘straight-liners’) were identified as invalid and were disregarded (N = 10). These invalid records were identified by visually searching through the data for records with the same responses throughout the survey. This process can be retraced in the file ‘Survey_speeders_straightliners_highlighted.xlsx’ in the folder ‘3. Processing/Adjustments’. We did not specify a cut-off on response times because case-by-case evaluation of the data by a researcher was more informative about the response quality. Section ‘Methods – Processing steps’ further provides what corrections were done to enhance the quality of the responses.

The proxies for residential proximity to the coast are based on residential postal codes, and not on accurate coordinates or addresses. As such, the user should keep in mind that the linear, shortest, and fastest travel routes reported in the data probably differ to a certain degree from the real routes.

## Usage Notes

Familiarization with the survey design, questions, response options, and processing steps performed is encouraged before interpretation, exploration and analysis of the data. After familiarization, the final processed dataset can be explored and analysed with the desired statistical software at any difficulty level to answer any of the research questions proposed in the introduction or other ones. Ideally, the survey weights are to be considered during the analyses, and the scope in time (i.e. 2022) and space (i.e. Flemish inhabitants and visits to the Belgian coast) should be respected during interpretation.

## Data Availability

The software R (R-4.3.0, RStudio 2023.03.1 + 446)^66^ was used for processing the data. The R script can be found within the data^45^ on location ‘/3. Processing/R workspace/ Hooyberg_SurveyCoastalVisits_2022-08-31.R’).
